# Miscellaneous Cases and Observations

**Published:** 1843-05

**Authors:** J. S. Sawyer

**Affiliations:** Vincennes, Indiana


ART. III.—*Miscellaneous cases and observations.* Read before the Vincennes Medical Society, November, 1842. By DR. J. S. SAWYER, of Vincennes, Indiana.

On the 9th May, 1841, Mrs. E. was delivered of a delicate female child exhibiting the natural appearances, except a large ventral hernia. This tumour, about two and a half inches in diameter, at its base, occupied the lower and anterior portion of the symphisis pubis. The surface presented a fiery red appearance, and was covered with small elevations, resembling fungous excrescences, at the side of each of which was a small puncture. The whole tumour discharged an irritating, ichorous fluid. Its size varied with the different degrees of distention of the bowels. Fearing either ulceration or gangrene, I directed the most soothing applications, and the most scrupulous care to prevent chafing.

This being, to me at least, a novel case, I requested my friend Dr. Posey to see it, who agreed with me that nothing could be done at that time for permanent relief. As I had apprehended, the surface soon began to ulcerate, and in a short time so large an opening was formed, that a great portion of the bladder was protruded through it. It now appeared evident that the abdominal parietes were entirely wanting, and that the hernial sac itself was exposed. As to the precise nature of the connexion of the tumour at its circumference, with the integuments of the abdomen, I cannot hazard an opinion. The child during the month contracted the infection of pertusis, which, together with the irritation of the hernia, soon carried it off. A post-mortem examination would have been desirable, and might have shown the upper portion of the pubes to have been wanting. But owing to the delicate health of the mother it was not proposed.

The complaints arising from inflammation and irritation of the spinal cord, have of late attracted much attention and been the subject of many dissertations. Like most new things this discovery has no doubt been overrated, and spinal irritation has been made to account for the production of ailments not easily explained *otherwise*—just as we denominate many complaints and symptoms nervous, which we do not understand. Believing however, that spinal irritation does not receive undue attention in this region, I shall relate a case or two which may serve to show some of the anomalous symptoms that may be produced by it. In the fall of 1840, I was called upon by a lady of my acquaintance aged about thirty-five and requested to prescribe for her. She stated that for several days she had been unable to speak above her breath, felt general debility, and particularly, a weakness of the upper extremities. She had had several attacks of this kind before, (which, like the present, had appeared without any apparent cause), for which digitalis had been prescribed and taken without much benefit. The patient's constitution had been much shattered, and she had had some years before, an attack of hæmoptysis. I prescribed an expectorant mixture of syrup of squills, antimonial wine, and elixir paregoric, which affording no relief, the patient in a short time called again. It now occurred to me that the aphonia might depend upon spinal irritation and a consequent partial paralysis of the nerves supplying the organs of voice. Finding, upon examination, great tenderness of the lower cervical and one or two of the upper dorsal vertebræ, I prescribed a blister, which had scarcely drawn before complete relief was obtained. Nearly two years have since elapsed, but there has been no return of the complaint. Sometime afterwards, I attended a lady who was subject to attacks of aphonia and weakness of the upper extremities, upon making any unusual muscular exertion. The same condition of the spine was found to exist, and the same treatment was successful as in the other case. It may be well to state that this lady has since had an attack of hæmoptysis.

It is well-known, that the phenomena of voice depend upon the internal laryngeal, and the recurrent nerves, which are branches of the par vagum, and are distributed, the former to the epiglottis and muscles moving the cartilages, and the latter to the larynx and trachea. The spinal accessory, which with the glosso-pharyngeal, and par vagum makes up the eighth pair, takes its origin from twigs of the fourth, fifth, sixth, and seventh cervical nerves, and after uniting with the other two divisions of the eighth, is finally distributed to the muscles of the shoulder. This connexion seems to explain the production of aphonia, pain in the shoulder, and weakness of the upper extremities, from an irritation of the spine at the origin of the spinal accessory nerve.

Inflammation and congestion of the brain, prevailed to an alarming extent in some parts of the country, during the last winter. A striking feature of this epidemic, was the rapidity of its progress. I shall attempt to give some account of these complaints as they occurred within the circle of my practice, not so much with a view of saying anything new, as with the hope of stimulating other members to communicate the result of their observations and reflections. In the early part of February last, I was called to visit a boy aged about six years, the son of a gentleman who had already lost another child, after a few hours illness. He complained of great soreness of the limbs and the whole surface of the body. Lying in bed produced great uneasiness and pain, and when I attempted to examine his pulse he would cry out with anguish. The surface was most of the time cool, though in some part of the day, there was slight reaction. The skin was covered with small, smooth, dark-red points, not much larger than a pin's head. This was a case which would no doubt have been called at one time, cold-plague; and Saalmon in his account of epidemic *inflammation* of the *brain*, says, that it sometimes made its appearance with imperfect reaction, and great pain and soreness of the limbs. Considering a strong tendency to *congestion* of the brain manifested in this case, I prescribed the semicupium, blisters to the extremities, and a large dose of calomel. I did not see the patient again, but understood that the calomel did not operate for twenty-four hours, when a large quantity of black bilious matter was discharged, with considerable amendment of the symptoms. This case might have recovered under a vigorous course of counter-irritation, and evacuation from the liver, but having no further treatment, it went on to a fatal termination. Before I left the house, the infant in the cradle was observed to be more fretful than usual. A simple prescription was made, hoping that it was not seriously indisposed, but in about thirty-six hours it expired.

In a few days I was requested to visit a boy about four years old, in the same neighborhood. I arrived about two o'clock, P. M. I was informed that he had not appeared indisposed until the morning of the same day, when he complained of his head, and growing rapidly worse, the family sent for aid. When I arrived the patient was nearly insensible. The surface was rather cool, and covered with small smooth points of a purple color—pulse small and weak. The eyes had a vacant appearance, and no impression was made upon the pupil by the approach of a candle. Considering the rapid progress of the case, and the deeply congested state of the brain, I could not give the parents any encouragement. The patient however was immersed in a warm bath, which produced great prostration. Blisters and sinapisms were then applied to the extremities and stomach, a large dose of calomel was, with much difficulty, administered, and another directed to be given in two hours if practicable. The progress of the disease was not at all arrested, and, in twenty-six hours from the time when he first complained, the patient was a corpse. Just before I left, the youngest child, probably ten months old, was observed to be fretful; but I was not asked to prescribe, and did not examine it. In six hours it died. I do not know what were the symptoms, but suppose they were the same as in the other case. The case here related I consider one of congestion of the brain; a disease which, so far as I know, is of much more rapid progress than inflammation, properly so called, of that organ.

I shall briefly relate a case of inflammation of the brain which came under my care in the latter part of March. The patient was a little girl about five years old. She had been complaining some days before I was called, but was not considered dangerous until Thursday, when she became so much deranged as to alarm the family and induce them to send for aid. I arrived about two o'clock, P. M. The little patient could now seldom be made to answer a question, and when she did, the answer was altogether incoherent. The face was flushed, the eyes red and suffused, the pupil dilated. She would frequently rise in bed as if much affrighted, muttering inarticulately. The hands were tremulous, and the whole features indicated great nervous excitement. Vomiting, one of the usual symptoms in this complaint, was present in this case. About a dozen large worms had been thrown from the stomach. The pulse was weak and frequent. In view of the rapid progress of the case, the difficulty of administering medicine, and of the fact that the second stage of the disease was already present, I considered this case as hopeless. I however administered a large portion of calomel and rhubarb, and left several others to be given two hours apart if possible; and a strong preparation of pink and senna was directed to be given in as large doses and as often as practicable. Blisters were applied to the temples and extremities. In the evening the medicine had not operated—directed it to be continued through the night when practicable. The vomiting continued at intervals all night, and the medicine, notwithstanding the use of injections, did not operate until eleven o'clock next day, when large dark evacuations were had, together with twenty-six large worms in one knot. By this time, however, the patient was rapidly sinking, and expired about one o'clock, less than twenty-four hours from the time when I first saw her.

I propose to close these imperfect remarks with a few reflections. Inflammation and congestion of the brain, though they may arise from the same cause, yet obviously do not consist in the same condition of the brain. In other words, like causes do not produce like effects, in all systems and at different times. The most common cause of both in this climate, is doubtless cold. This cause may, in one individual, produce inflammation in the substance of the brain or its membranes, ending, unless arrested by timely depletion, in gangrene or effusion; while in another, so great a shock is given to the nervous system that no re-action takes place, and the powers of life seem at once to succumb. Whether in congestion of the brain, the condition of that organ is essentially inflammatory, I shall not presume to decide. I believe not. In general we understand a congestion of any part to consist in an over-distention of the vessels of that part, and a sluggish motion of the contained fluid, *without* inflammation. There is no doubt that the direct effect of cold is to weaken the action of the heart. This principle, so easy of demonstration if necessity required and time permitted it, will be taken for granted, in attempting an explanation of the proximate cause of congestion of the brain. The heart being weakened in its action, the blood must necessarily accumulate in the right side of the heart and in the large veins leading to it, for the reason that the organ has not power to free itself from the burden, by filling the arteries. The blood being, consequently, imperfectly arterialized, does not exercise its natural amount of stimulus upon the heart, which tends indirectly still further to weaken its action. This state of congestion occurs to a great or less extent in the cold stage of every paroxysm of fever, but in these cases, *reaction* coming on, restores the balance of the circulation. The causes which tend to render the amount of congestion relatively greater in one organ than another, may be various; as constitutional or accidental weakness of the part, predisposition, &c. In the operation of any cause weakening, in a great degree, the action of the heart and producing consequent venous congestion, the *brain*, returning as it does so large a quantity of blood to the right aurich, *must* to a very great extent be implicated in that congestion. But when cold produces such inroads upon the nervous system, as to prostrate at once the vital energies, so that reaction cannot take place, it is but natural that the *brain*, which is the *centre* of that system, should be the principal seat of congestion. This tendency, so natural in itself, may be increased by any source of irritation in other parts, the effects of which are most naturally transmitted by sympathy of parts to the brain. I am persuaded that in the epidemic of last winter, this source of irritation was the presence of worms. I am convinced from observation and reflection, that this cause, by producing an undue excitement in the brain, hastened the progress of the disease and rendered the fatal result more certain. Indeed, there is reason to suspect that in some cases of the kind, worms are the main cause, and the application of cold, merely a concomitant. So well am I persuaded of the mischief they produce in many affections of the brain, that I consider their expulsion as one of the first indications, in the early stage of the complaint.

It may be useful to attempt a diagnosis between inflammation and congestion of the brain. *Inflammation* is known by the general heat of the skin, the flushed countenance, the red eye, pain, generally acute in some part of the head, vomiting, early delirium, and contracted pupil. The pulse is said to be sometimes full, but generally hard and small. In *congestion*, the reaction is feeble, and in the worst cases there is none; consequently, there is little or no external heat. The face, instead of being flushed, is *pale*, or has a *darker* hue than natural. Instead of the delirium and nervous agitation, there is languor, coma, soon followed by insensibility. The pulse is weak, soft, and compressible. The period of insensibility occurs much sooner than in inflammation. There is reason to believe that the immediate cause of death in congestion, is not, as in inflammation, the process of effusion or gangrene, but the compression of the brain from over distention of its vessels or from their actual rupture.

With regard to the treatment of these complaints few words will suffice. Where they occur in a violent form, as in the epidemic of last winter, nothing less than the most vigorous course of treatment in the very onset of the disease, can be of any avail. The misfortune is that, not apprehending danger, the friends do not call a physician until the disease has made much progress; when, even if he could always have the necessary means at hand, the stage for active depletion is past. Where however the patient can be seen in time, there can be no doubt of the absolute necessity of the abstraction of blood, though in the cases of children it must be done cautiously, as it is admitted that they do not bear blood-letting with equal impunity as adults. In inflammation, bleeding from the arm and cupping seem to answer the indication; but in congestion I think bleeding from the arm contra-indicated. Cupping the temples and nape of the neck, and opening the jugular vein appear to afford the only rational means of relief, so far as blood-letting is concerned. The next step is to remove all causes of irritation, and of these in cases of children the most important is worms. Ten grains of calomel should be given to a child five years old, in bad cases, repeated every three hours until it operates. A strong preparation of pink and senna should follow the first portion of calomel, and be repeated every hour in as large doses as the patient will swallow, until free evacuations have been procured from the whole. There should be no waiting or hesitating in this course, for when worms are present proving a source of irritation, unless sufficient medicine be administered *before stupor occurs*, to operate freely in a short time, the case will inevitably go on to a fatal termination. The calomel is always indicated, because, when it acts upon the liver producing black discharges, it derives powerfully from the head, and when worms are present it is *doubly* required. Even when no worms are expelled, the calomel, pink and senna are beneficial, because they seem to quiet them, and, operating briskly, these medicines produce the timely removal of other offending matter from the stomach and bowels. We dare not wait for the slow operation of these medicines given in the usual way, though they act more surely. A few hours brings stupor, when it is very difficult to give medicine in sufficient doses. Besides, the free operation of medicine after that time seems to hasten the fatal result. Blisters should be applied at an earlier period than is usual in affections of the head. In all doubtful cases, if blisters are used, it should be done immediately upon the appearance of a disposition to coma. If delayed, they will frequently not act at all. In inflammation of the brain which runs its usual course, it is better to defer their application until the violence of the disease is somewhat abated.

